# Books and Magazines

**Published:** 1907-11

**Authors:** 


### Books and Magazines.

Business Methods of Specialists, or How the Advertising Doctor Succeeds.—An exposition of the inside workings of the complicated structure the advertising specialist has built about himself, the doors of which are seldom opened to the professional investigator. By Jacob Dissingeo Albright, M. D., editor of Albright's Office Practitioner, author of the General Practitioner as a Specialist, a Treatise on Legitimate Medical Specialties. Published by the author. Philadelphia, 1907. Cloth, $1.25, postpaid.

The character and scope of this little book are set forth in the above. The regular medical profession has never before had an opportunity of obtaining real, reliable inside information regarding the advertising specialist's methods, and I feel sure my readers will be interested to know that this book is available.

Progressive Medicine.—A quarterly digest of advances, discoveries and improvements in the medical and surgical sciences. Edited by Hobert Amory Hare, M. D., Professor of Therapeutics and Materia Medica, Jefferson Medical College, Philadelphia. Lea Bros. & Co., Philadelphia and New York. $6 per annum.

We have the September number (Vol. III) of this valuable series. It contains contributions on the following subjects by the most distinguished authors: "Diseases of the Thorax and Its Viscera, Including the Heart, Lungs and Bloodvessels," by William Ewart, M. D., F. R. C. P.; "Dermatology and Syphilis," by William S. Gottheil, M. D.; "Obstetrics," by Edward P. Davis, M. D.; "Diseases of the Nervous System," by William G. Spiller, M. D. This number is an exceptionally valuable one.

---

A neuralgic pain in the region of the ear should suggest a careful examination of the teeth for a possible caries or alveolar abscess.—*American Journal of Surgery.*

